# Supplementary material for: Brain control of bimanual movement enabled by recurrent neural networks
Source: Sci Rep. 2024 Jan 18;14:1598. doi: 10.1038/s41598-024-51617-3 (PMC10796685; doi:10.1038/s41598-024-51617-3)
Supplement: Supplementary file 1 — Supplementary Information 1. [file 41598_2024_51617_MOESM1_ESM.pdf]

Brain control of bimanual movement enabled by recurrent neural networks

Darrel R. Deo[1,2], Francis R. Willett[4], Donald T. Avansino[4], Leigh R. Hochberg[7-10], Jaimie M. Henderson[1,2,11†], and Krishna V. Shenoy[2-6,11†]

[†]These authors contributed equally to this work

Supplementary Figures & Tables

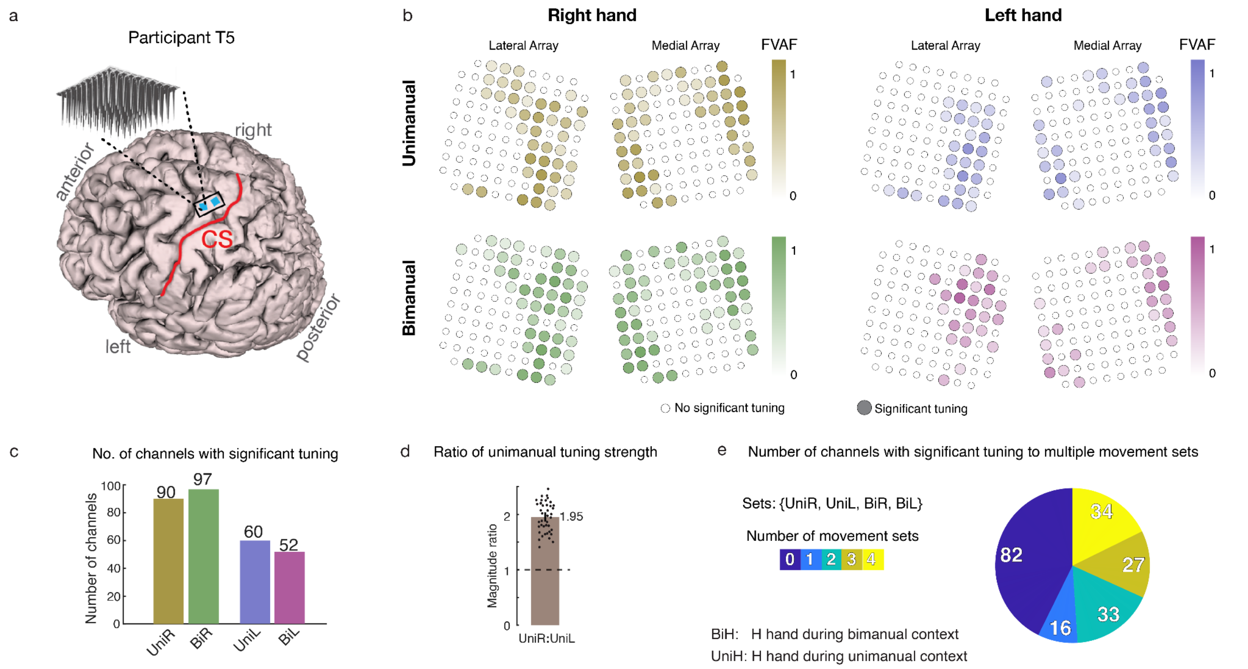

Supplementary Fig. 1 | Tuning to unimanual and bimanual movement is intermixed within electrodes and has no clear somatotopic pattern. a Participant T5's MRI-derived brain anatomy and microelectrode array locations. Microelectrode array locations were determined by co-registration of post-operative computed tomography (CT) images with preoperative MRI images. b The strength of each electrodes' tuning to right or left hand movement during unimanual and bimanual movement contexts is indicated with a shaded color (darker colors indicate more tuning). Tuning strength was quantified as the fraction of total firing rate variance accounted for by changes in firing rate due to the movement conditions (unimanual/bimanual). Small white circles indicate electrodes that had no significant tuning to that movement context as governed by a 1-way ANOVA. Broad spatial tuning to all movement categories can be seen across all arrays. c Bar plots indicate the number of electrodes that were significantly tuned to each movement context as computed in (a). Results show greater preference for right hand tuning across both movement contexts. d Ratio of unimanual tuning strength between the right and left hand. Tuning strength was computed using an unbiased estimate of neural distance between tuning coefficient vectors. The right hand had almost twice as strong tuning than the left hand. e Pie chart summarizes the number of electrodes that had statistically significant tuning to each possible number of movement sets (from 0 to 4).

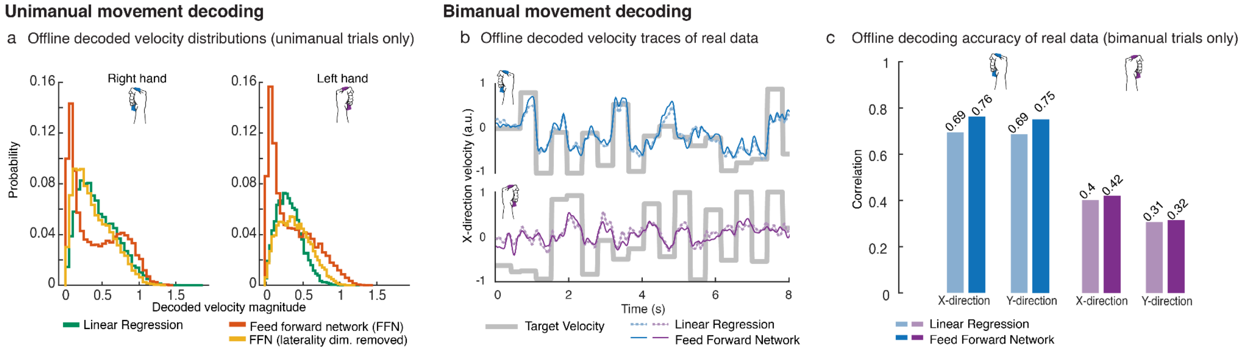

Supplementary Fig. 2 | Offline unimanual and bimanual decoding. a Distributions of decoded velocity magnitudes during unimanual movement (related to Fig. 3a,b). The feed forward neural network (FFN) was able to decode higher velocity magnitudes than the linear decoder. Removal of the laterality dimension resulted in less decoded velocities near 0 for the FFN, indicating more cursor jitter without laterality information. **b** Offline single-bin decoding on bimanual data. Neural activity was binned (20-ms bins) and truncated to 400 ms movement windows (300-700 ms after go cue). Linear ridge regression (RR) and a densely connected FFN (single layer, 512 units) were trained, using 5-fold cross-validation, to decode left and right cursor velocities. Sample 8 s snippets of decoded x-direction velocity traces are shown. **c** Each bar indicates the offline decoding performance (Pearson correlation coefficient) for the RR and FFN decoders across the x- and y-direction velocity dimensions. Generally, right hand decoding accuracy was higher than left hand decoding accuracy during bimanual movement.

a RNN architecture

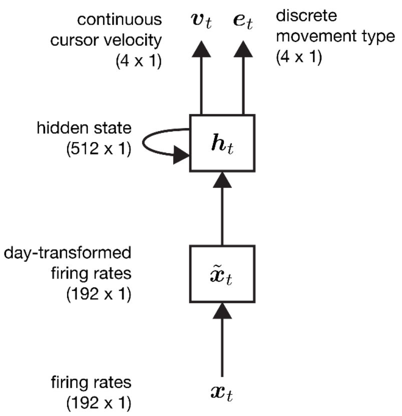

Supplementary Fig. 3 | Diagram of the RNN architecture. a We used a single-layer RNN with 512 gated recurrent units (GRUs; h_t) to transform neural firing rates (x_t) binned in 20 ms to continuous cursor velocities (v_t) and discrete movement signals (e_t). The v_t vector describes the x- and y-direction velocities for the right (first two dimensions) and left (last two dimensions) cursors at that moment in time (t), and e_t is a one-hot vector (only one dimension is high at any given time) which codes for the type of movement that the RNN detects (unimanual right, unimanual left, bimanual, or no movement) at that time point. Note that we used a day-specific affine transform on the input firing rate vector x_t to account for day-to-day changes in neural activity.

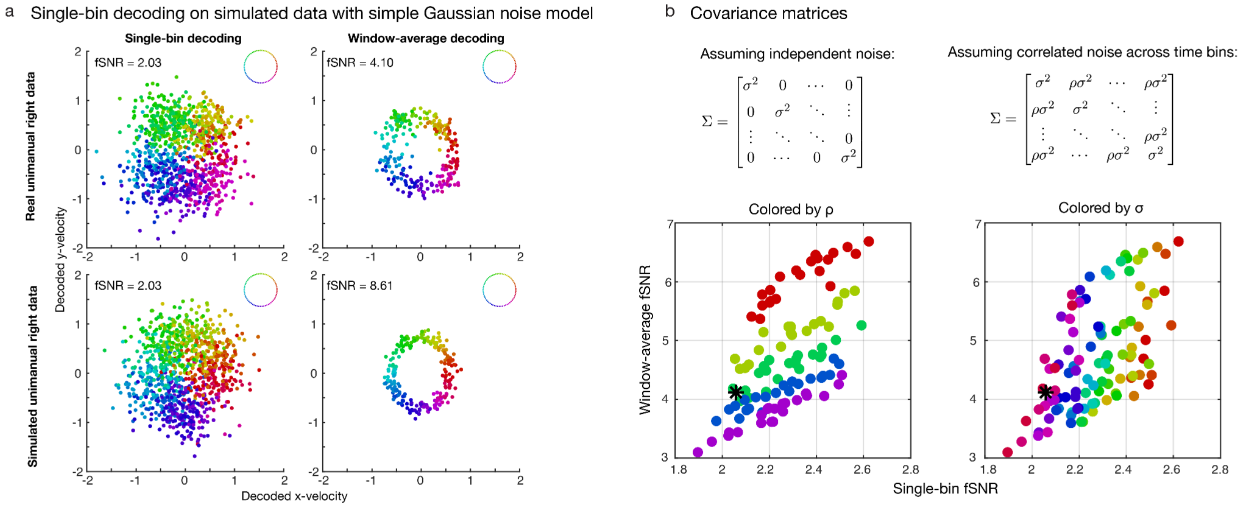

Supplementary Fig. 4 | Decoding simulated data with a simple Gaussian noise model. a Using a functional signal-to-noise ratio (fSNR; see Methods) metric, we quantify single-bin and window-average decoding performance on real data (top row) and simulated data (bottom row). The simulated data was created using a simple Gaussian noise model (using the left covariance matrix in b) which assumes independence between firing rates across time bins for any given electrode channel. Decoders were built using cross-validated linear regression on 20-ms binned data in the movement window from 300 to 700 ms after the go cue of each trial. The single-bin decoders were calibrated on each 20-ms bin of data, whereas the window-average decoders were calibrated on the averaged activity within the 400-ms window. Each dot represents the decoded x- and y-direction velocity in either each 20-ms time bin (left column) or each 400-ms window of a trial (right column). The color of each dot corresponds to the true target direction of movement indicated by the keys in the upper right of each panel. In this example, the simulated data was generated to match the single-bin fSNR of real unimanual right hand movement data (2.03). Notice that although the single-bin fSNRs of both the real and simulated datasets match, the window-average fSNRs differ quite significantly. **b** The simple gaussian model on the left assumes independent noise, whereas the covariance matrix on the right assumes correlated noise across time bins. σ is the standard deviation, and ρ is the correlation coefficient. **c** In order to match the window-average fSNR, one could use the correlated noise model and sweep the covariance parameters until a window-average fSNR is met. The scatter plots indicate the single-bin and window-average fSNRs of synthetic datasets created by sweeping a range of both σ and ρ parameters in the Gaussian model with correlated noise. Black stars indicate the real data's single-bin and window-average fSNR as seen in panel a. Both plots are identical except for the way in which the points are colored. The plot on the left is colored according to the ρ value, and the plot on the right is colored by the σ value. Notice that the correlated noise (ρ parameter) mainly affects the window-average fSNR and the single-bin SNR is mainly affected by the standard deviation parameter σ. With our focus on single-bin decoding, the simple Gaussian noise model was sufficient when generating synthetic datasets.

X-direction	UniL-BiL	UniR-UniL	BiR-BiL
**UniR-BiR**	**7.82×10^{-10}**	**4.89×10^{-40}**	**2.39×10^{-11}**
**UniL-BiL**		**3.4×10^{-21}**	0.05
**UniR-UniL**			**1.04×10^{-12}**

Y-direction	UniL-BiL	UniR-UniL	BiR-BiL
**UniR-BiR**	**3.3×10^{-9}**	**9.32×10^{-27}**	**4.0×10^{-16}**
**UniL-BiL**		0.76	**3.28×10^{-6}**
**UniR-UniL**			**8.51×10^{-13}**

Supplemental Table 1 | Two-sample T-tests for significance between tuning correlations. These p-values correspond to the bar plots in Figure 2c. Bolded entries indicate significance as any value below 0.01. BiH denotes H hand during the bimanual context, and UniH denotes H hand during the unimanual context.

Date	Session #	Trial day	Description	Figure / Movie
06.02.2021	320	1750	Cued unimanual and bimanual hand movement	Fig 1B-C<br>SFig 1B,C,E
06.04.2021	321	1752	Cued unimanual and bimanual hand movement (pilot data used to initially calibrate RNNs for closed-loop)	Fig 5A-C
06.23.2021	324	1771	Cued unimanual and bimanual hand movement (pilot data used to initially calibrate RNNs for closed-loop)	Fig 5A-C
06.28.2021	325	1776	Cued unimanual and bimanual hand movement, closed-loop two-cursor control	Fig 2B-D<br>Fig 5A<br>SFig 1D
06.30.2021	326	1778	Cued unimanual and bimanual hand movement, closed-loop two-cursor control	Fig 2B-D<br>Fig 5A<br>SMovie 1
07.12.2021	329	1790	Cued unimanual and bimanual hand movement, closed-loop two-cursor control	Fig 5A
07.14.2021	330	1792	Cued unimanual and bimanual hand movement, closed-loop two-cursor control	Fig 2B-D<br>Fig 5A
09.13.2021	336	1853	Cued unimanual and bimanual hand movement, closed-loop two-cursor control (RNN vs. linear regression)	Fig 5C
09.15.2021	337	1855	Cued unimanual and bimanual hand movement, closed-loop two-cursor control (RNN vs. linear regression), 'Unimanual task' variant	Fig 5C<br>SMovie 3
09.27.2021	340	1867	Cued unimanual and bimanual hand movement, closed-loop two-cursor control (augmented vs. non-augmented training data)	Fig 4E
09.29.2021	341	1869	Cued unimanual and bimanual hand movement, closed-loop two-cursor control (augmented vs. non-augmented training data)	Fig 4D - E<br>SMovie 4
10.11.2021	344	1881	Cued unimanual and bimanual hand movement, closed-loop two-cursor control (sequential unimanual vs. simultaneous bimanual strategy)	Fig 2B-D<br>Fig 5A-B
10.13.2021	345	1883	Cued unimanual and bimanual hand movement, closed-loop two-cursor control (sequential unimanual vs. simultaneous bimanual strategy)	Fig 2B-D<br>Fig 5A-B<br>Fig 3A-C<br>SMovie 2<br>SFig 2A-C

Supplemental Table 2 | List of data collection sessions with participant T5.

Supplemental Movie 1 | Simultaneous bimanual control of two cursors via RNN decoding. In this movie, participant T5 uses a BCI to control two cursors in real-time to targets on a computer monitor. An RNN converts neural activity into velocities for both cursors at each timestep. On each trial, one of three movement types are cued randomly: (1) bimanual (simultaneous movement of both cursors), (2) unimanual right (only right cursor movement), or (3) unimanual left (only left cursor movement). Each trial begins with a 'prepare' segment (of random duration) where lines connect each cursor to its intended target. T5 prepares to move during this segment but does not attempt movement until the lines disappear, indicating the 'go' cue. Successful target acquisition occurs when both cursors simultaneously dwell within their designated target (illuminates blue) for an uninterrupted period of 0.5 s. A trial times out at a maximum of 10 s. The RNN decoder is enabled at all times. This experiment block was recorded during a performance evaluation session reported in Figure 5a (trial day 1778).

Supplemental Movie 2 | Sequential unimanual movement vs. simultaneous bimanual movement. The same as Supplemental Movie 1, except T5 uses two different movement strategies: (1) sequential unimanual (moving one cursor at a time), and (2) simultaneous bimanual (moving both cursors simultaneously). A separate RNN decoder is used for each movement strategy. The RNN used for the simultaneous bimanual strategy is trained normally (just like in supplemental video 1) with both unimanual and bimanual data. The RNN used for the sequential unimanual strategy is trained only with unimanual trials. Both experiment blocks were recorded during a performance evaluation session reported in Figure 5b (trial day 1883).

Supplemental Movie 3 | RNN vs. linear decoder for two-cursor control. The same as Supplemental Movie 1, except with only unimanual trials. An RNN decoder is compared to a linear decoder for online control of two cursors. This task was limited to unimanual trials to focus on the differences between decoders. Both experiment blocks were recorded during a performance evaluation session reported in Figure 5c (trial day 1855).

Supplemental Movie 4 | Online two-cursor control with raw and temporally altered training data. Same as Supplemental Movie 1, except with only unimanual trials. During this task, one cursor is cued on any given trial where the other cursor stays 'locked' in place. This version of the task was used to focus on the differences between decoders. One decoder was trained with raw training data and the other decoder was trained with temporally altered training data. Both experiment blocks were recorded during a performance evaluation session reported in Figure 4e (trial day 1869).
